# Direct Regeneration of Degraded LiFePO_4_ Cathode via Reductive Solution Relithiation Regeneration Process

**DOI:** 10.3390/molecules29143340

**Published:** 2024-07-16

**Authors:** Chenchen Li, Rui Gong, Yingjie Zhang, Qi Meng, Peng Dong

**Affiliations:** 1 Faculty of Metallurgy and Energy Engineering, Kunming University of Science and Technology, Kunming 650093, China; lichenchen@stu.kust.edu.cn (C.L.); gongrui2017@163.com (R.G.); zhangyingjie09@126.com (Y.Z.); 2 National and Local Joint Engineering Laboratory for Lithium-Ion Batteries and Materials Preparation Technology, Key Laboratory of Advanced Battery Materials of Yunnan Province, Kunming University of Science and Technology, Kunming 650093, China

**Keywords:** lithium iron phosphate, spent cathode materials, direct regeneration, hydrothermal reduction

## Abstract

The rapid growth of electronic devices, electric vehicles, and mobile energy storage has produced large quantities of spent batteries, leading to significant environmental issues and a shortage of lithium resources. Recycling spent batteries has become urgent to protect the environment. The key to treating spent lithium-ion batteries is to implement green and efficient regeneration. This study proposes a recycling method for the direct regeneration of spent lithium iron phosphate (LFP) batteries using hydrothermal reduction. Ascorbic acid (AA) was used as a low-cost and environmentally friendly reductant to reduce Fe^3+^ in spent LiFePO_4_. We also investigated the role of AA in the hydrothermal process and its effects on the electrochemical properties of the regenerated LiFePO_4_ cathode material (AA-SR-LFP). The results showed that the hydrothermal reduction direct regeneration method successfully produced AA-SR-LFP with good crystallinity and electrochemical properties. AA-SR-LFP exhibited excellent electrochemical properties, with an initial discharge specific capacity of 144.4 mAh g^−1^ at 1 C and a capacity retention rate of 98.6% after 100 cycles. In summary, the hydrothermal reduction direct regeneration method effectively repairs the defects in the chemical composition and crystal structure of spent LiFePO_4_. It can be regarded as a green and effective regeneration approach for spent LiFePO_4_ cathode materials.

## 1. Introduction

Over the past few decades, lithium-ion batteries (LIBs) have been extensively used in electric vehicles (EVs), communication base stations, large-scale energy storage, and other industries due to their high voltage, high energy density, and long cycle life [1,2]. According to statistics, global EV sales were 550,000 in 2015, which increased to 3.1 million by 2020, with an average annual growth rate exceeding 40% [3]. Lithium iron phosphate (LFP) batteries are renowned for their high storage capacity, excellent thermal stability, minimal toxicity, and cost-effectiveness, rendering them extensively employed in the battery systems of mainstream EVs [4,5,6]. However, given the typical service life of 5 to 8 years for LFP batteries, their retirement phase has arrived. It is estimated that by 2025, the cumulative quantity of spent LFP batteries will reach 4 × 105 tons [7,8]. Additionally, spent LFP batteries contain various toxic and harmful substances, including electrolytes, organic adhesives, and conductive agents, which can cause significant environmental pollution and ecological harm [9,10]. Furthermore, battery production accounts for 65% of the world’s lithium (Li) resource utilization [11]. Recycling spent batteries will prevent the waste of these resources, aligning with the principles of carbon neutrality and sustainable development.

Currently, the recovery and reuse of spent LFP batteries primarily involve pyro-/hydro-metallurgical recycling (pyro-/hydro-) and direct regeneration [12]. The pyro-process generally adopts high temperatures to obtain lithium salt vapor and metal oxide as a simple and mature strategy for industrial application [13]. However, this approach is accompanied by high energy consumption and greenhouse gas emissions. On the other hand, typical processes for hydro-regeneration mainly involve leaching [14], separation [15], and purification [16]. Leaching methods extensively studied in recent years include acid [17], salt [18], roasting [19], bioleaching [20], and electrochemistry [7]. Nevertheless, it fails to prevent the excessive depletion of acid/alkali reagents in a cumbersome recycling step [21]. The processes often require a substantial quantity of raw materials and are accompanied by the generation of residues containing heavy metals, thereby resulting in secondary pollution, which is inconsistent with the principles of green chemistry and sustainable development [13]. Moreover, due to the low content of the high-value component (Li) in the LFP cathode, the economic value of the recycled product cannot be realized through the costly pyro-/hydro-metallurgical techniques alone.

In recent years, direct regeneration has restored the electrochemical capacity of spent LFP materials [22,23]. The direct regeneration technique involves the direct lithium replenishment of spent LFP materials by physical and chemical methods to restore their electrochemical performance [24]. The electrochemical capacity degradation of LFP materials after long-term cycling is mainly attributed to the loss of free Li deposited in the anode and solid electrolyte interface (SEI) film [25]. Unlike layered cathode materials, the cell volume change of LFP materials during charging and discharging is very small. The framework of the olivine structure of the LFP materials does not collapse, which is beneficial for the direct repair and regeneration process of the spent LFP materials [26,27]. The current direct regeneration studies can be mainly categorized into solid-phase and liquid-phase regeneration [28]. High-temperature calcination induces lithium ions to diffuse back into the lithium vacancy gaps of the olivine structure, enabling the regeneration of spent cathode materials with high crystallinity and ideal stoichiometric ratios. However, the traditional high-temperature solid-phase direct regeneration method has the limitation of being inconsistent with the waste conditions. Liquid-phase regeneration is a method to regenerate degraded materials by replenishing lithium using the liquid phase. The method does not require the precise addition of lithium due to the self-limiting nature of lithium reduction in aqueous solutions and the omission of stoichiometry in hydrothermal methods. It also consumes less energy due to milder temperature conditions and shorter reaction times. The high-valence transition metals in spent batteries are usually insoluble in water [29], necessitating the addition of a reducing agent to achieve efficient leaching. Previous studies have frequently utilized organic acids as reducing agents due to their biodegradability, low cost, and mild reaction conditions, with examples including oxalic acid, pyruvic acid, and malic acid. However, a crucial factor often overlooked is that a lower ionization potential (IP) corresponds to a stronger reducing ability. Organic acids possess significantly lower IPs compared to inorganic acids. For instance, ascorbic acid (AA) has a lower IP than citric acid, acetic acid, and oxalic acid [22,29,30] (Figure S1). Therefore, this study explores the use of ascorbic acid as a reducing agent, a topic that has been scarcely reported in the literature.

This study proposes a feasible strategy of using ascorbic acid as a reducing agent to replenish lithium by hydrothermal reduction on spent LFP materials to prepare regenerated LFP materials. The role of ascorbic acid in the hydrothermal process and its effect on the electrochemical properties of the regenerated LFP materials were mainly investigated. And the regenerated LFP materials have good recycling properties and rate properties. Therefore, this economical and green method is expected to be used for large-scale recycling of spent LFP materials in the future.

## 2. Results and Discussion

### 2.1. Structural and Morphology Characterization

**Figure 1:**
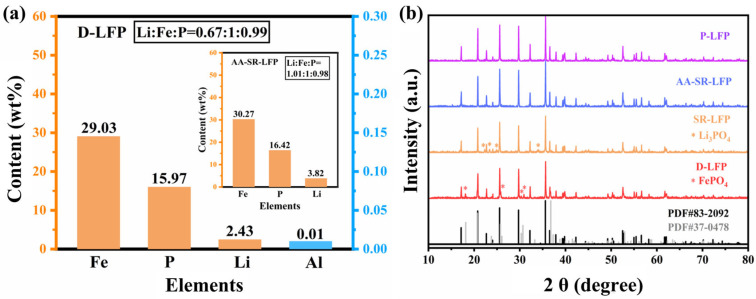
(**a**) Element content of spent LFP (D-LFP) and regenerated materials (AA-SR-LFP); (**b**) XRD patterns of four materials, D-LFP, AA-SR-LFP, P-LFP, and SR-LFP.

ICP-OES was tested to ascertain the content of metal elements in spent LFP materials (D-LFP) and regenerated materials (AA-SR-LFP), as illustrated in Figure 1a. The Li/Fe molar ratio in D-LFP is 0.67, which indicates that there was a significant loss of the Li element in the spent LFP materials. This is due to the inevitable occurrence of phenomena such as the lithium dendrite, continuous formation of solid electrolyte interface (SEI) film, and irreversible phase transitions in both cathode and anode electrodes during long-term cycling of LiFePO_4_ batteries, leading to Li loss and capacity decay [31]. However, in the restorative material (AA-SR-LFP), the ratio of Li to Fe reaches 1.01 (Figure 1a). According to the XRD pattern in Figure 1b, there are FePO_4_ impurity peaks present at 18.2°, 30.3°, and 30.7° in the D-LFP materials. This is due to irreversible phase transitions occurring during prolonged charge–discharge cycles of LFP batteries [22], leading to the coexistence of LiFePO_4_ and FePO_4_ phases in the long-cycled LFP [32]. XRD results show that the structure of D-LFP material has been destroyed, which is consistent with the ICP analysis.

In the XRD pattern of SR-LFP, the FePO_4_ peak disappeared, but the Li_3_PO_4_ peak emerged. This suggests that Li ion (Li^+^) does not effectively diffuse into the interior of the material particles during hydrothermal regeneration to repair the crystal structure of D-LFP. The diffraction peaks of AA-SR-LFP and P-LFP align with the standard diffraction peaks of olivine-type LiFePO_4_ crystal and exhibit excellent crystallinity. In conclusion, AA can effectively transform the impurity phase FePO_4_ in the spent LFP materials into LiFePO_4_. Thus, lithium replenishment through hydrothermal reduction can achieve the restoration of the crystal structure of spent LFP materials.

**Figure 2:**
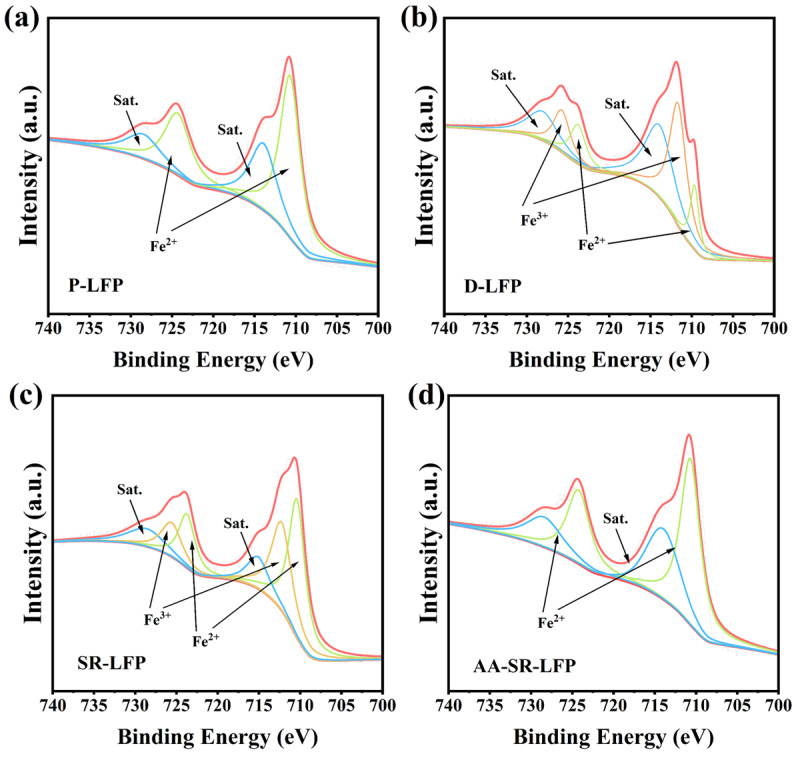
XPS analysis of Fe 2p regions of (**a**) P-LFP, (**b**) D-LFP, (**c**) SR-LFP, and (**d**) AA-SR-LFP.

Figure 2 shows the Fe 2p pattern of the XPS analysis of P-LFP, D-LFP, AA-LFP, and AA-SR-LFP. The Fe 2p spectrum exhibits two distinct signals attributed to Fe 2p_3/2_ and Fe 2p_1/2_, arising from spin-orbit coupling. Each signal comprises a main peak and a “vibrational” satellite peak. The peak splitting results of Fe 2p in D-LFP (Figure 2b) indicate the presence of both Fe^2+^ and Fe^3+^ in the materials. Fe^3+^ arises from the irreversible impurity phase FePO_4_ generated during the long-term charge–discharge cycles of LFP batteries [33]. It is noteworthy that only Fe^2+^ peaks are present in P-LFP and AA-SR-LFP, indicating that during the reductive hydrothermal reaction process, Fe^3+^ in FePO_4_ is reduced to Fe^2+^ and combines with Li^+^ in the solution to form LiFePO_4_. This confirms the significance of AA in facilitating the reduction of Fe^3+^ to Fe^2+^, promoting the reinsertion of Li^+^ into the LFP cathode material to compensate for charge, thus achieving the restoration of the crystal structure of spent cathode electrode materials.

**Figure 3:**
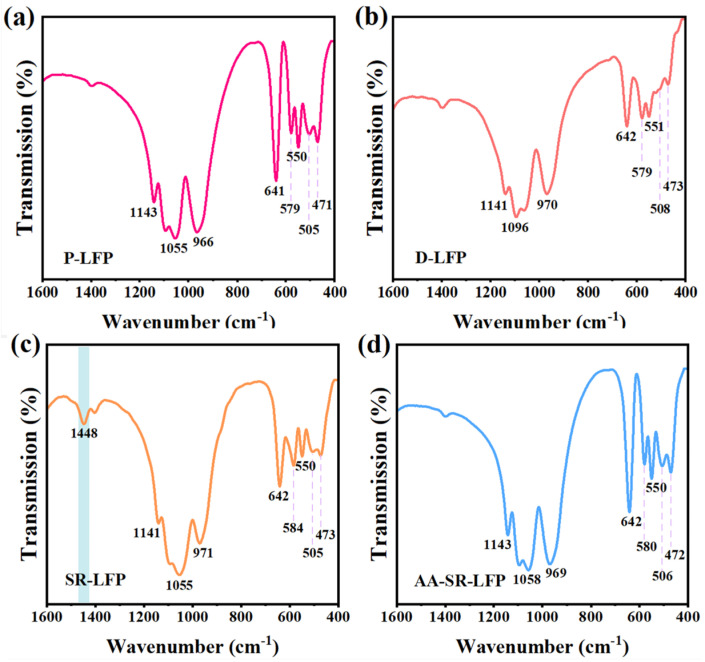
FTIR spectra of (**a**) for P-LFP, (**b**) for D-LFP, (**c**) for SR-LFP, and (**d**) for AA-SR-LFP.

Furthermore, the FTIR spectra were used for further analyzing the structural evolution of spent LFP material during direct regeneration technology operation and the stability of the functional groups, as shown in Figure 3. The essential feature of hydrothermal regeneration is the change in the valence and lattice parameters of Fe upon embedding Li^+^, leading to small changes in the vibrational frequencies of the FTIP spectra. Due to the presence of impurity of phase FePO_4_, the antisymmetric stretching vibrational absorption peak of the P-O bond in the PO_4_^3−^ group in LiFePO_4_ shifts from 1055 cm^−1^ to 1096 cm^−1^ from in D-LFP (Figure 3b). The absorption peaks at 641 cm^−1^, 578 cm^−1^, and 551 cm^−1^ in the fingerprint region correspond to the bending vibrational absorption peaks of the FeO_6_ octahedron and PO_4_ tetrahedron in LFP, showing little deviation, whereas the vibration of AA-SR-LFP (Figure 3b) is stronger than that of D-LFP and SR-LFP (Figure 3c). Notably, SR-LFP exhibits a characteristic peak at 1448 cm^−1^, which is not observed in the other samples. Combined with the XRD results, this may correspond to a certain vibrational region of Li_3_PO_4_. Moreover, the vibrational bands at 505 cm^−1^ and 471 cm^−1^ are associated with the motion of Li^+^, with their intensity decreasing as x decreases in Li_1−x_FePO_4_. Overall, the hydrothermal lithium replenishment directly regenerates the crystal structure, without disrupting the olivine lattice framework composed of FeO_6_ octahedra and PO_4_ tetrahedra.

Figure S4 represents the SEM micrographs of the P-LFP (a), D-LFP (b), SR-LFP (c), and AA-SR-LFP (d) samples, showing no significant difference. This indicates that the hydrothermal lithium replenishment direct regeneration method did not alter the original morphology of the materials. The particles of D-LFP (Figure S4b) samples exhibit uneven distribution and a rough surface, with a significant agglomeration phenomenon observed without clear boundaries. This agglomeration leads to material particle non-uniformity, reducing the material’s packing density and hindering Li^+^ transport, thereby decreasing Li^+^ deintercalation efficiency [22,34]. Conversely, the AA-SR-LFP (Figure S4d) particles appear as bright and clear overall, with a reduction in the agglomeration phenomenon. This could be attributed to the high-pressure environment during the hydrothermal reaction, causing D-LFP particles to disintegrate and disperse from the secondary agglomerates.

**Figure 4:**
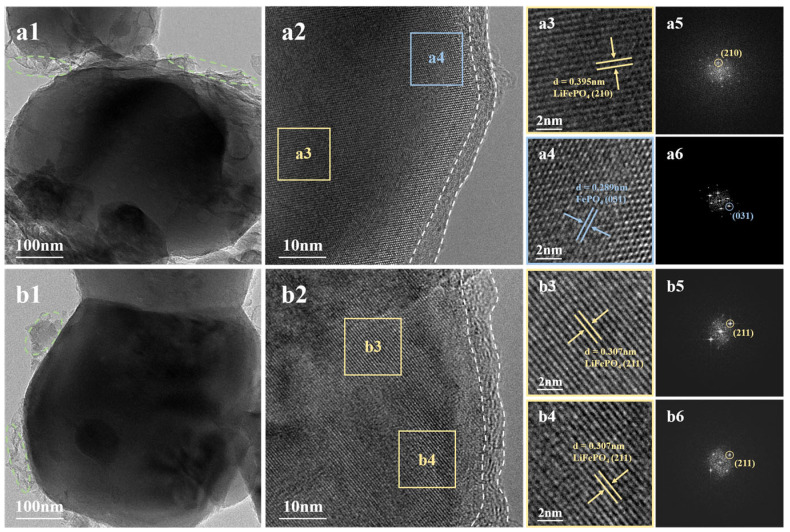
HRTEM images of D-LFP (**a1**–**a4**) and AA-SR-LFP (**b1**–**b4**); the FFT pattern of D-LFP (**a5**,**a6**) and AA-SR-LFP (**b5**,**b6**).

One of the key reasons for the degradation of cathode materials is the change in crystal structure [35]. Therefore, D-LFP and AA-SR-LFP materials were subjected to the HRTEM analysis to further elucidate the evolution of the crystal structures, as depicted in Figure 4. A small amount of amorphous material was observed on the surfaces of both D-LFP and AA-SR-LFP, Figure 4(a1,b1), which may be residual trace conductive carbon. Notably, it can be observed that a carbon coating with a thickness of about 5 nm was retained on the surface of AA-SR-LFP in Figure 4(a2,b2). This indicates that the lithium embedding process does not affect the micro-morphological structure of the material.

As can be seen in Figure 4(a2), different crystal structure regions are observed in D-LFP. The spacing of the lattice stripes was measured to be 0.395 nm in the enlarged view of the region in Figure 4(a3), which is consistent with the (210) crystal plane of LiFePO_4_ (PDF#83-2092). And the corresponding diffraction pattern was obtained by FFT transformation (Figure 4(a5)). Similarly, the spacing of the lattice fringes was measured to be 0.289 nm in the enlarged view of the region in Figure 4(a4), which corresponds to the (031) plane of FePO_4_. The presence of FePO_4_ is attributed to the lack of Li^+^, induced by excessive lithium extraction and volume changes during charge–discharge cycles of the cathode electrode material, leading to the formation of lithium vacancies in the olivine structure [36]. In the crystal structure of spent cathode electrode materials, the presence of numerous impurity phases and crystal defects greatly impedes the diffusion of lithium ions [37]. This is a significant reason for the poor cycling and rate performance of spent cathode electrode materials.

However, Figure 4(b2–b4) shows that after regeneration, only the (211) crystal plane of LiFePO_4_ can be observed in the AA-SR-LFP material. All lattice fringes exhibit a uniform direction and consistent lattice spacing of 0.307 nm. This was confirmed by FFT transformation (Figure 4(b5,b6)) to be the (211) lattice plane of orthorhombic olivine LiFePO_4_. The HRTEM analysis indicates that the hydrothermal reduction lithiation regeneration method can convert FePO_4_ lattice planes into LiFePO_4_ in the D-LPF material without altering the morphology and microstructure, thereby repairing crystal structure defects.

**Figure 5:**
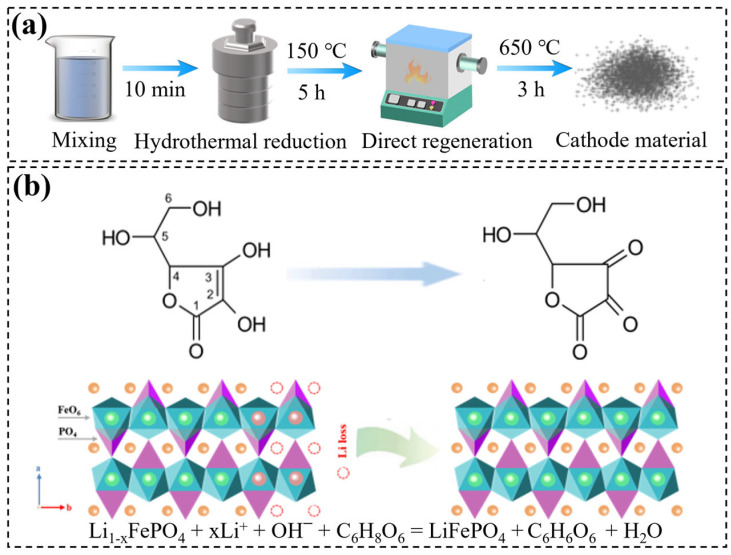
Hydrothermal reduction lithium regeneration flow chart: (**a**) for flow chart of experiment; (**b**) for regeneration mechanism diagram.

Overall, the D-LFP particles contain irreversible phase transformation products, specifically FePO_4_, but their olivine structure remains largely intact, exhibiting only lithium deficiency. During the hydrothermal reduction lithiation regeneration process, Fe^3+^ in the impurity phase FePO_4_ is reduced to Fe^2+^, forming a negatively charged olivine structure. Concurrently, Li^+^ in the solution fills the lithium vacancies through thermal diffusion, achieving lithiation and repairing crystal structure defects, thereby yielding the regenerated cathode material AA-SR-LFP. The reaction equation for hydrothermal reduction lithiation regeneration is shown in Figure 5b.

### 2.2. Electrochemical Performance

**Figure 6:**
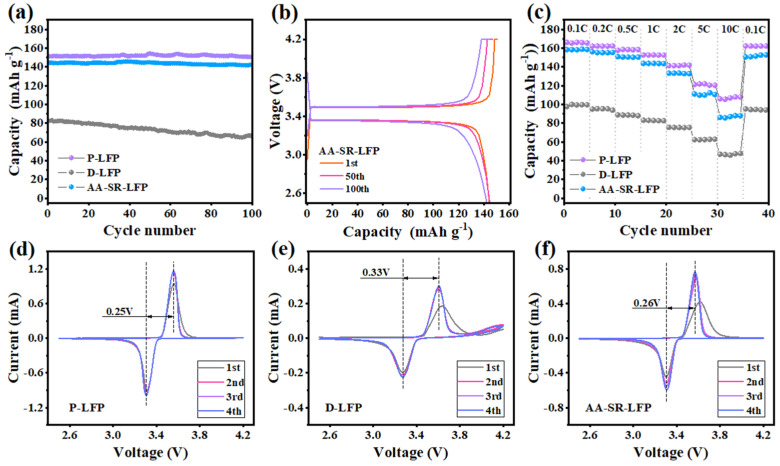
Cyclic performance of the three materials (**a**). (**b**) For the charge–discharge curves of AA-SR-LFP during the 1st, 50th, and 100th cycles. (**c**) For the rate performance of the three materials. (**d**) For cyclic voltammetry curves of P-LFP. (**e**) For cyclic voltammetry curves of D-LFP. (**f**) For cyclic voltammetry curves of AA-SR-LFP.

The multiple electrochemical performances (2.5–4.2 V) (1 C = 170 mA g^−1^) of commercial materials (P-LFP), spent LFP materials (D-LFP), and direct regeneration materials (AA-SR-LFP) were tested at room temperature, as shown in Figure 6. And the results show that the cyclic stability (1 C) from high to low is P-LFP, AA-SR-LFP, and D-LFP (Figure 6a). The initial discharge specific capacity of P-LFP reaches 151.3 mAh/g at 1 C, with a capacity retention rate of 99.5% after 100 cycles. AA-SR-LFP exhibited electrochemical performance very close to that of P-LFP, with 144.4 mAh/g and 98.6% retention, respectively. In contrast, D-LFP had an initial discharge capacity of only 82.5 mAh/g and 81.1% retention after 100 cycles at 1 C, much lower than P-LFP and AA-SR-LFP. The analysis of the ICP and XRD results indicates that the Li^+^ deficiency in D-LFP restricts its ion mobility during cycling, leading to its lower discharge capacity. The crystal structure of the material directly regenerated by hydrothermal reduction shows significant improvement, thus enhancing its electrochemical performance.

Figure 6b illustrates the charge–discharge curves of AA-SR-LFP during the 1st, 50th, and 100th cycles at a current density of 1 C within the voltage range of 2.5–4.2 V. Although the discharge plateau slightly shortens after 100 cycles, the discharge specific capacity remains almost unchanged, with a retention rate of 98.6%. Moreover, the Coulomb efficiencies for the 1st, 50th, and 100th cycles were 95.83%, 99.94%, and 99.85%, respectively, maintaining an exceptionally high value close to 100% throughout the 100 cycles at 1 C.

The rate performances of P-LFP, AA-SR-LFP, and D-LFP are demonstrated in Figure 6c. The D-LFP materials exhibit poor rate performance, with an initial discharge specific capacity of only 115.4 mAh/g at a current density of 0.1 C, and a low discharge specific capacity of just 40.1 mAh/g at 10 C, indicating severe damage to the crystal structure of the spent cathode material [38]. In contrast, the AA-SR-LFP demonstrates superior electrochemical performance, with discharge specific capacities of 158.3, 156.1, 150.7, 143.6, 133.2, 111.1, and 85.9 mAh/g at 0.1 C, 0.2 C, 0.5 C, 1 C, 2 C, 5 C, and 10 C, respectively, approaching the level of P-LFP. This is because the crystal structure of the sample is better restored, promoting L^+^ diffusion and thereby enhancing its electrochemical performance.

The charge–discharge curves of P-LFP, D-LFP, and AA-SR-LFP at different rates are depicted in Figure S5. The specific capacity of all samples decreased significantly as the charge–discharge rate increased. However, P-LFP and AA-SR-LFP maintained higher specific capacities and exhibited distinct charge–discharge plateaus. In comparison to D-LFP, AA-SR-LFP demonstrated superior charge–discharge specific capacity and a reduced gap between the charge–discharge curves, indicating lower electrochemical polarization and enhanced electrochemical reversibility of the regenerated material.

Cyclic voltammetry can be used to assess the reversibility of electrochemical reactions and phase transitions [39]. Cyclic voltammetry was performed on P-LFP, D-LFP, and AA-SR-LFP samples in the range of 2.5–4.2 V at a scan rate of 0.1 mV/s, as shown in Figure 6d–f. Figure 6e shows that the CV curve of spent LFP exhibits low repeatability, indicating relatively poor cycle stability. The cyclic oxidation–reduction peaks, corresponding to the phase transition reaction (LiFePO_4_/FePO_4_) of the lithium iron phosphate electrode material during charge and discharge, are attributed to the deintercalation of Li^+^ during the charge and discharge processes (redox reaction between Fe^2+^ and Fe^3+^). This phase transition is also the primary mechanism of operation for LFP batteries [40]. Specifically, the P-LFP (Figure 6d) exhibits the smallest polarization voltage (0.25 V) and the highest peak current. The redox peak difference between the Fe^2+^ and Fe^3+^ in D-LFP is 0.33 V, indicating a significant polarization effect in the spent LFP materials. The redox peak voltage difference in AA-SR-LFP is notably smaller than in D-LFP, at 0.26 V. Furthermore, the peak currents of AA-LFP are higher than those of D-LFP, indicating a larger enclosed area (higher discharge specific capacity). This observation is consistent with the charge–discharge curves. AA-SR-LFP shows excellent reversible redox peak positions and minimal polarization, suggesting good structural reversibility. These findings highlight the effectiveness of the hydrothermal reduction method in improving Li^+^ deintercalation and reversibility.

**Figure 7:**
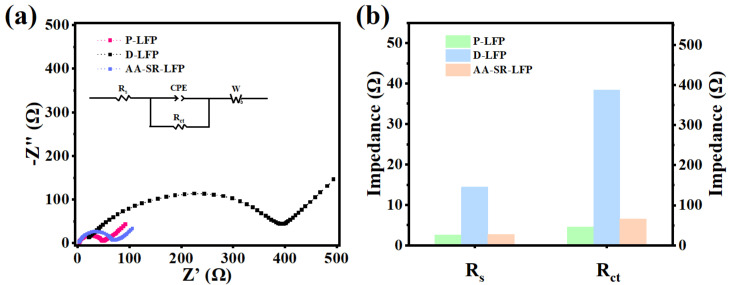
Electrochemical impedance spectroscopy profiles of P-LFP, D-LFP, and AA-SR-LFP. (**a**) for impedance curve; (**b**) for fitting value.

Figure 7 shows the electrochemical impedance spectra of P-LFP, D-LFP, and AA-SR-LFP measured by charging to 4.2 V at 1 C. The electrochemical impedance spectra consist of a flattened semicircle in the high-frequency region and a slanted straight line in the low-frequency region. Based on the migration of Li^+^ from the electrolyte to the bulk phase of the material, the electrochemical impedance spectra can be divided into two parts. Components such as capacitance and inductance are used to combine into an equivalent circuit, the equivalent circuit diagram is embedded in Figure 7a, and the fitting results of the impedance of each part are shown in Figure 7b. It is intuitive to see that the semicircular arc of the D-LFP is the largest, which corresponds to the fitted impedance values of 14.4 Ω and 388.0 Ω for Rs and Rct, respectively. It shows that the waste cathode material has high ohmic resistance and charge transfer impedance. It may be due to the thicker SEI film on the anode surface of the D-LFP material after thousands of cycles. As a result, the Li^+^ diffusion resistance is increased, which makes it difficult to cross the SEI film. The impedance values of the regenerated material are substantially reduced, with Rs and Rct values of 2.69 Ω and 66.5 Ω, respectively, for the AA-SR-LFP. It is only slightly larger than the value for the P-LFP, but very close. This indicates that the conductivity of the regenerated cathode material is substantially improved, and the smaller interfacial film impedance and charge transfer impedance are favorable for Li^+^ diffusion.

## 3. Experimental Section

### 3.1. Pretreatment of Materials

Pyro-/hydro- and direct regeneration processes always start with pretreatment (discharging and materials separation) [32,41]. The spent LFP batteries utilized in this experiment were sourced from BYD Company in China. First, spent LFP batteries were immersed in a 2 mol/L sodium sulfate (Na_2_SO_4_) solution for discharging. They were then disassembled to separate the cathode electrode, diaphragm, graphite anode, shell, and other components. A suspension of spent LFP cathode electrodes was obtained by ultrasonication in a 3 mol/L sodium hydroxide (NaOH) solution to separate the coating of the spent LFP materials from the cathode electrode. The suspension of spent LFP materials was filtered and washed with deionized water to remove residual sodium aluminate (NaAlO_2_) from the surface. Finally, the filter residue was dried at 80 °C for 12 h to obtain the spent LFP cathode materials, labeled as D-LFP.

### 3.2. Direct Regeneration of D-LFP

The regeneration process of the spent LFP battery cathode material is shown in Figure 5. About 4 g of cathode material powder (D-LFP) was added into a lithium hydroxide (LiOH) solution with ascorbic acid (AA) and stirred for 10 min to ensure homogeneity. The solution was transferred into a 100 mL hydrothermal reactor and placed in a constant temperature oven at 150 °C for 5 h for hydrothermal reduction and lithium replenishment. The filtered residue was vacuum-dried at 120 °C for 12 h. The dried material was annealed in a tube furnace under an argon atmosphere at 650 °C for 3 h. The obtained cathode material by hydrothermal reduction and lithium replenishment was labeled as AA-SR-LFP. In total, 4 g of spent cathode material powder (D-LFP) was directly added to a LiOH solution without AA, and treated similarly, and the cathode electrode materials obtained by hydrothermal lithium replenishment were labeled as SR-LFP for the control, while the commercial material was recorded as P-LFP.

### 3.3. Material Characterization

The cathode materials (AA-SR-LFP and SR-LFP) were dissolved by HNO_3_-HCl, and the elements’ contents of the solution were measured by inductively coupled plasma optical emission spectrometry (ICP-OES, Prodigy, Leeman, Hudson, NH, USA). The crystal structure of the samples was analyzed by X-ray diffraction (XRD, Rigaku Mini Flex 600, Tokyo, Japan). And the scanning angle (2θ) ranges from 10° to 90°, with a scanning speed of 5° min^−1^. Scanning electron microscopy (SEM, TESCAN-VEGA3, Shanghai, China) and high-resolution transmission electron microscopy (HRTEM, TECNAI G2 TF30 S-TWIN, Amsterdam, The Netherlands) were used to further study the micro-morphology and lattice structure of the samples. Changes in valence states of elements and the material composition of the samples before and after regeneration were analyzed using X-ray photoelectron spectroscopy (XPS, Thermo Fisher Scientific, Waltham, MA, USA, K-Alpha^+^), and Fourier Transform Infrared Absorption Spectroscopy (FTIR) was used to test the samples.

### 3.4. Electrochemical Measurements

Electrochemical performance tests were conducted to demonstrate the superiority of the regenerated materials. In an agate mortar, mix the cathode material, polyvinylidene fluoride (PVDF), and acetylene black in a mass ratio of 8:1:1 uniformly. The volumetric bottle was then filled with N-methyl-2-pyrrolidone (NMP), which was then thoroughly agitated with a magnetic stirrer at 400 rpm/min to create a homogenous slurry. The slurry was evenly coated onto the surface of aluminum foil using an automatic coating machine, followed by vacuum drying at 120 °C for 8 h. The half-cell assembly is conducted in an inert gas-filled glove box with a water oxygen content of <0.05 ppm. Place the electrode in the center of the cathode shell, then add the electrolyte (1 M LiPF_6_ dissolved in a solvent mixture of EC:DEC:PC = 1:1:1). Subsequently, sequentially position the separator, lithium metal foil, nickel mesh, and anode shell, and finally use a button cell sealing machine for compression sealing.

Utilize the LAND (CT2001A, Wuhan, China) testing system to conduct constant galvanostatic charge–discharge tests on the assembled half-cell. The test temperature is maintained at room temperature and the cut-off voltage ranges from 2.5 to 4.2 V. The nominal capacity at 1 C is 170 mAh/g. The cyclic voltammetry (CV) test voltage range is 2.5–4.2 V with 0.1 mV/s scanning speed. The electrochemical impedance spectroscopy (EIS) test is operated in Auto-Lab. The frequency range is 0.1 Hz–10^5^ Hz and the amplitude is 1 mV.

## 4. Conclusions

In this study, LFP cathode materials with excellent crystallinity and electrochemical properties were successfully regenerated by the hydrothermal reduction direct regeneration method. The results of ICP and XRD analyses revealed issues with D-LFP, including lithium deficiency and heterogeneity in FePO_4_ distribution. The hydrothermal regeneration of AA-SR-LFP in a lithium-reduced solution with ascorbic acid restored the stoichiometric ratio of Li to Fe and the crystal structure of the original material. XPS and FTIR analyses, along with characterization results, revealed that nearly all of the Fe^3+^ in the cathode material, regenerated directly by hydrothermal reduction, was converted to Fe^2+^. SEM and HRTEM analyses demonstrated the crystal structure of the spent LFP cathode material, without any problems. Furthermore, they showed that the original morphology of the particles remained intact after the crystal structure repair. These findings strongly support the feasibility of direct lithium replenishment through hydrothermal reduction. The electrochemical data measured at 2.5–4.2 V indicate that the regenerated material AA-SR-LFP exhibits superior electrochemical performance compared to the spent cathode material. It achieves an initial discharge specific capacity of 144.4 mAh g^−1^ at 1 C, with a capacity retention rate of 98.6% after 100 cycles. Overall, the hydrothermal reduction method effectively restores the defects in the chemical composition and crystal structure of the spent LFP cathode material. This cost-effective and environmentally friendly approach holds promise for large-scale recycling of spent LFP cathode materials in the future.

## Data Availability

The data presented in this study are available on request from the corresponding author. The data are not publicly available due to specific ethical and privacy considerations.

## Supplementary Materials

The following supporting information can be downloaded at: https://www.mdpi.com/article/10.3390/molecules29143340/s1.
