# Supplementary material for: Direct Regeneration of Degraded LiFePO4 Cathode via Reductive Solution Relithiation Regeneration Process
Source: Molecules. 2024 Jul 16;29(14):3340. doi: 10.3390/molecules29143340 (PMC11279840; doi:10.3390/molecules29143340)
Supplement: Supplementary file 1 [file molecules-29-03340-s001.zip › molecules-3072575-supplementary.docx]

**Direct Regeneration of Degraded LiFePO_4_ Cathode via
Reductive Solution Relithiation Regeneration Process**

Chenchen Li^1,2^, Rui Gong^1,2^, Yingjie Zhang^1,2^, Qi Meng^1,2*^, Peng Dong^1,2**^

^1^Faculty of Metallurgy and Energy Engineering, Kunming University of Science and Technology, Kunming 650093, China

^2^National and Local Joint Engineering Laboratory for Lithium-ion Batteries and Materials Preparation Technology, Key Laboratory of Advanced Battery Materials of Yunnan Province, Kunming University of Science and Technology, Kunming 650093, China

* Corresponding author.

E-mail addresses: [mengqi315117@126.com](mailto:mengqi315117@126.com). (Qi Meng)

[dongpeng2001@126.com](mailto:dongpeng2001@126.com) (Peng Dong)


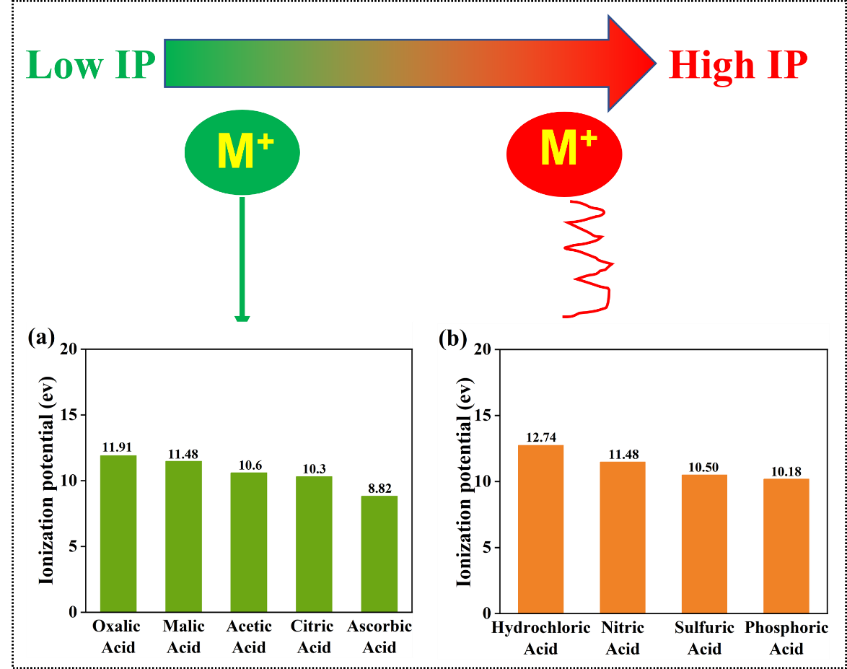


**Figure S1.** (a) for ionization potential of organic acids, (b) for ionization potential of inorganic acids.

For the hydrothermal reduction process, we conducted experiments at temperature gradients of 80, 100, 120, 150, and 180 degrees Celsius to investigate the lattice structure restoration of regenerated materials from waste under different temperature conditions, as shown in **Fig. S2**. The results indicate that the optimal lattice restoration occurs at 150°C, while further increasing the temperature to 180°C does not significantly improve the restoration. Therefore, we selected 150°C for our experiments.


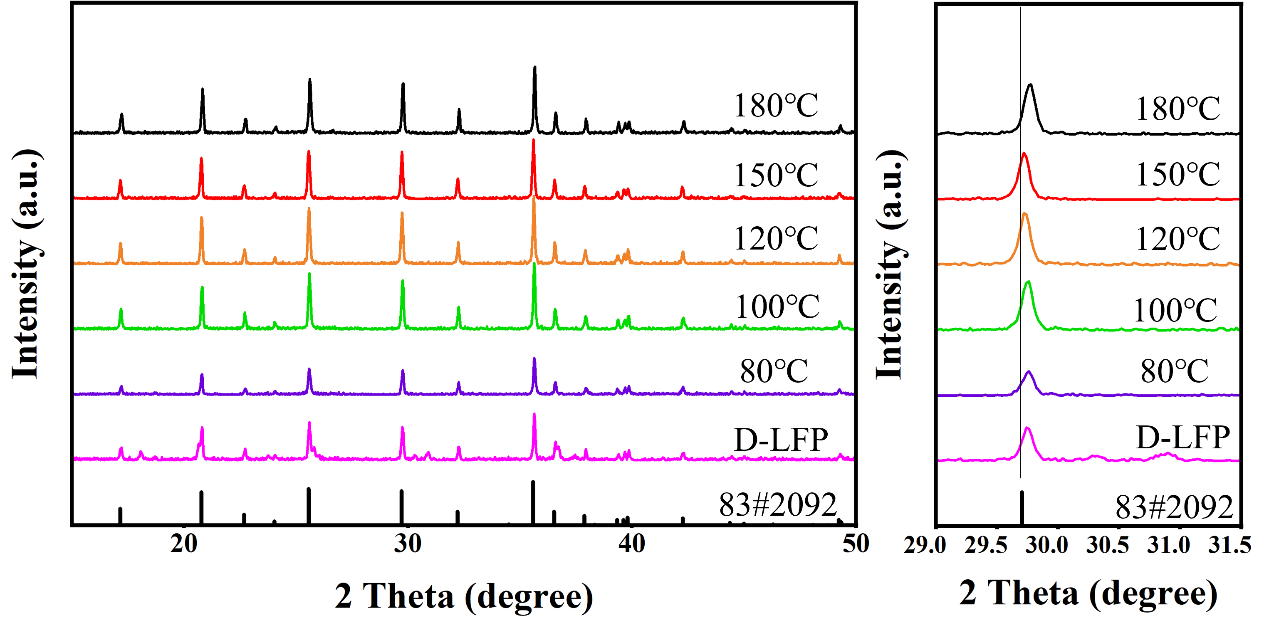


**Figure S2.** Hydrothermal reduction temperature exploration experiment.

**Fig. S3** presents the XRD patterns of regenerated cathode materials under hydrothermal conditions of 150°C for 5 hours with varying concentrations of ascorbic acid and lithium hydroxide. As shown in **Fig. S3a**, the diffraction peaks of regenerated cathode materials do not match the standard and exhibit impurity peaks at an ascorbic acid concentration of 0.3 mol/L under excessive lithium ion concentrations, indicating that lower concentrations are insufficient for restoring the crystal structure of spent cathode materials. When the ascorbic acid concentration increases from 0.3 mol/L to 0.9 mol/L, the diffraction peaks of the regenerated cathode materials align well with the standard. At a concentration of 1.2 mol/L, the diffraction peaks almost match the standard. These results suggest that an increased amount of reductant within a certain range aids in compensating for lithium loss.

**Fig. S3b** shows the XRD patterns of regenerated cathode materials at different Li+ concentrations. Similarly, the diffraction peaks of all regenerated cathode materials align well with the standard, and a Li+ concentration of 0.05 mol/L suffices to meet the theoretical lithium requirement. Increasing the Li+ concentration has no significant impact on the regenerated materials. Clearly, the missing lithium can be successfully replenished even at theoretical lithium dosages.


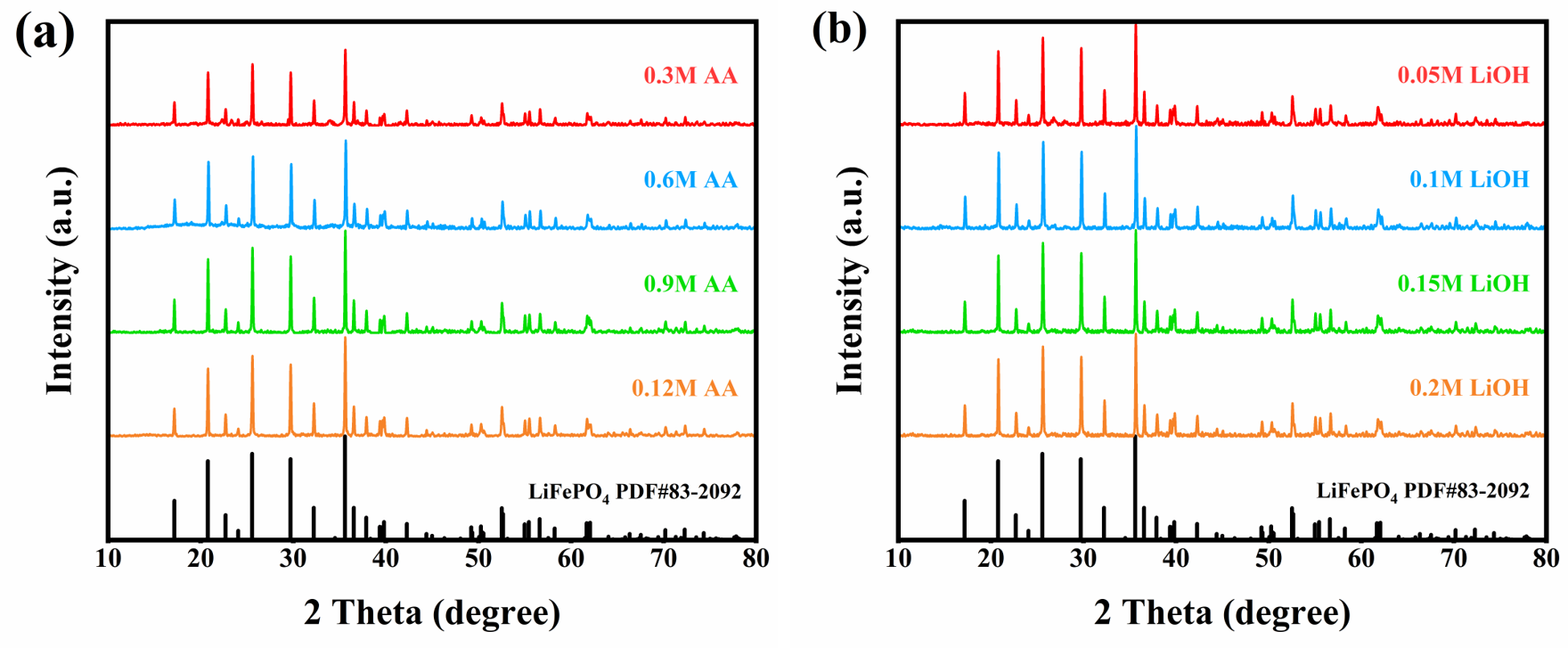


**Figure S3.** XRD patterns of regenerated cathode materials with different ascorbic acid and lithium hydroxide concentration


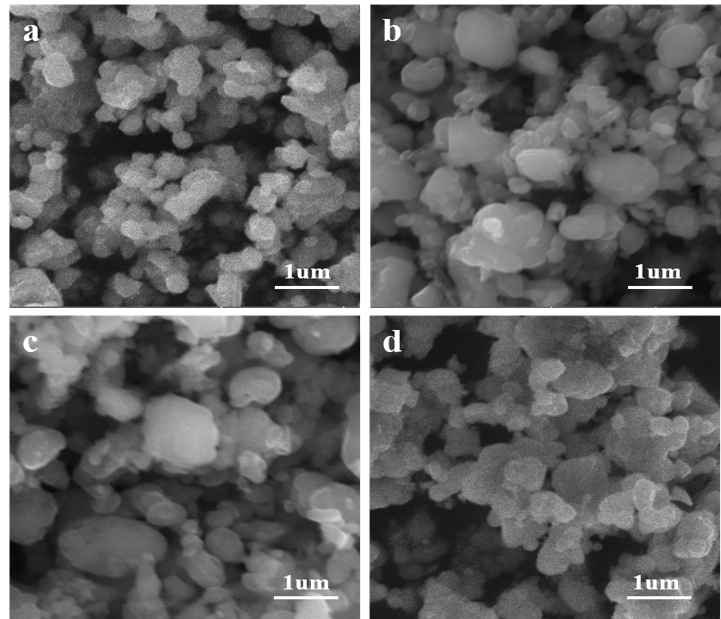


**Figure S4.** SEM images of four samples (a) for P-LFP, (b) for D-LFP, (c) for SR-LFP, (d) for AA-SR-LFP.


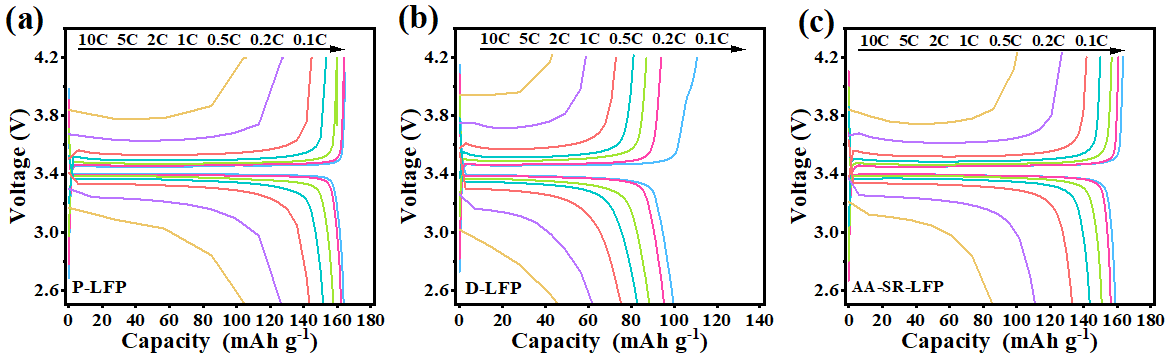


**Figure S5.** The first charge discharge curves of the sample at different rates: (a) for P-LFP, (b) for D-LFP, (c) for AA-SE-LFP.
